# Mold Contamination of Untreated and Roasted With Salt Nuts (Walnuts, Peanuts and Pistachios) Sold at Markets of Tabriz, Iran

**DOI:** 10.5812/jjm.8751

**Published:** 2014-01-01

**Authors:** Abdolhassan Kazemi, Alireza Ostadrahimi, Fereshteh Ashrafnejad, Nafiseh Sargheini, Reza Mahdavi, Mohammadreza Farshchian, Sepideh Mahluji

**Affiliations:** 1 Liver and Gastrointestinal Diseases Center, Tabriz University of Medical Sciences, Tabriz, IR Iran; 2 Nutritional Research Center, Faculty of Nutrition, Tabriz University of Medical Sciences, Tabriz, IR Iran; 3 Department of Biochemistry and Nutrition, Faculty of Nutrition, Tabriz University of Medical Sciences, Tabriz, IR Iran; 4 Department of Health of Environment, Tabriz University of Medical Sciences, Tabriz, IR Iran

**Keywords:** Fungi, Nuts, Contamination, *Aspergillus*

## Abstract

**Background:**

Nuts are one of the main consumed snacks worldwide and a significant component of Iranian’s diet. Natural contamination of nuts with fungus is unavoidable and is a major challenge to nuts safety and quality.

**Objectives:**

The purpose of this research was to study fungal contamination in commercially available nuts (pistachios, walnuts and peanuts) in the markets of Tabriz, Iran.

**Materials and Methods:**

100 samples of 50 gr roasted with salt peanuts and pistachios and 300 samples of 50 gr pure pistachios, walnuts and peanuts were collected from different areas of the local markets. After initial preparation, the samples were cultured on Sabouraud’s dextrose agar (SDA). 19 fungal isolates were identified.

**Results:**

The results show that *Aspergillus niger* was the predominant mold among pure (44%) and roasted with salt (14%) nuts (P < 0/001). In addition, percentage of mycotoxigenic fungal contamination was 18% for roasted with salt nuts and 11% for pure samples.

**Conclusions:**

The overall results of the analysed samples showed that the rate of fungal contamination in pure samples was higher than roasted with salt ones (P < 0.005). Results of the current survey could be useful for minimizing fungal contamination and can educate people about the dangers of mold in nuts.

## 1. Background

Fungi which produce several metabolites develop rapidly on different kinds of food and food products (1). These microorganisms can easily spread by wind, insects and rain (2). So far scientific studies have shown that various fungal species as much as 100000 are regarded as typical contaminants of food and agricultural crops (3). Molds naturally yield a wide range of metabolites which are called mycotoxins. Mycotoxins can cause toxic effects on human and animal tissue and organs (4). They are among the 21st century major concerns due to their important pathogenic role (5, 6). According to the previous studies, A. *Rhizopus* and *Penicillium* spp. are common molds in nuts, dried fruits and foodstuff (7, 8). 

Fungal contamination in nuts due to *Penicillium* spp., *Aspergillus*, *Fusarium* spp., *Trichoderma* spp. and *Cladosporium* spp. have been reported from America, Brazil and western Africa (9, 10). Food contamination with *Aspergillus* was first reported for pepper and then for other foodstuff such as nuts (11). An Iranian study indicated that 30% of pistachio and 36.1% of peanut products were contaminated with *Penicillium* spp. and *Aspergillus,* respectively (1). Various epidemiologic studies have indicated that fungus, especially species that produce aflatoxin, cause human gastrointestinal disorders, hepatic neoplasm, and liver cell carcinoma (4, 12). These days prevention of fungal contamination in foodstuffs especially nuts has become a public health issue (13). 

In some Asian and African countries, 30.97 million tons of the greasy products especially peanuts and pistachios are spoiled by *A. flavus* and *A. niger* (1). Fungal infestation happens during or after harvesting, storage and transition (14). Some important factors such as storage temperature, amount of moisture, oxygen existence and composition of gaseous compounds can affect the growth of mold during storage (15). However, several countries have tried to control the levels of mycotoxins, particularly aflatoxin in food and agricultural products, but sometimes it is difficult to deal with certain factors to lower levels of aflatoxin (16). In some countries such as Iran, production and consumption of nuts (untreated and roasted with salt) has increased (17, 18). Since nuts are known as a healthy food and have a pleasant taste, people have a great tendency to consume them instead of other snacks such as chips and popcorn (19).

## 2. Objectives

Fungal contamination is currently regarded as a public health concern and there is a global trend to reduce the resulting health problems. Moreover, considering the fact that nuts consumption is high in Iran (1), the aim of this study was to determine the contaminant microflora of Iranian nuts such as untreated and roasted with salt pistachios, peanuts and also untreated walnuts.

## 3. Materials and Methods

### 3.1. Samples

During the fall of 2011, 100 samples of each untreated nuts (pistachios, walnuts and peanuts) and 50 samples of each roasted with salt nuts (pistachios and peanuts) were collected randomly (area sampling) from deferent parts of the city (50 gr), transported in sterile packets to the laboratory and kept in a cool place (3 - 5ºC) for a maximum of three days. Nuts were surface-sterilised in 4% sodium hypochlorite for 2 minutes, diluted with distilled water three times to reach a concentration of 2%, rinsed in 100 mL distilled water and then let dry.

### 3.2. Mold Identification

Nuts were cultured on Sabouraud’s 4% dextrose agar (SDA, Merk, Germany) prior to incubation at 25ºC for 7 - 15 days and then examined daily for fungi growth. Taxonomic identification of fungi colonies was carried out by morphological, macro and microscopic characteristic, according to standard methods (20, 21). When necessary, culturing was repeated using other mediums (Czapel-Dex Agar (Oxoid - CM97, UK), Malt extract Agar (Oxoid - L39, UK) or APFA Agar (Oxoid- CM0731, UK)) for exact identification.

### 3.3. Statistical Analysis

For statistical analysis SPSS version 11.5 (SPSS Institute Inc. Chicago, Illinois) was used. Descriptive statistical analysis including mean ± SD for fungi number, frequency and percentage for determination of fungal contamination were used. Comparison of percentages was done by statistics calculation software that was downloaded from www.statpac.com.

## 4. Results

*A. niger *and *Mucor *were the predominant molds observed among the roasted with salt and untreated nuts. In addition, in the untreated group, *Penicillium *spp. (25.3%), *Helminthosporium *spp. (20%) and *Acremonium *spp. (15%) were prevalent and in the roasted group *A. fumigatus *(14%) was found in considerable amounts. Bacterial contamination in the roasted group (31%) was more than the untreated group (3%). According to Table 1, fungal contamination in the untreated group was higher than the roasted group. 

**Table 1.** Frequency Distribution (Count %) of Fungi Isolated From Different Nut Products

Fungi	Roasted With Salt, No. (%)	Untreated, No. (%)
***Aspergillus* spp.**	35 (35)	163 (54.3)
***Mucor^a^***	14 (14)	188 (62.7)
***A. niger^a^***	14 (14)	132 (44)
***A. fumigatus^a^***	13 (13)	8 (2.7)
***Penicillium spp.^a^***	8 (8)	76 (25.3)
***A. flavus***	5 (5)	18 (6)
***A. ochraceus^a^***	2 (2)	0
***Helminthosporium spp.^a^***	3 (3)	60 (20)
***Acremonium spp.^a^***	2 (2)	45 (15)
***Gliocladium* spp.**	1 (1)	9 (3)
***Trichoderma* spp.**	2 (2)	6 (2)
***Geotrichum Candidum***	3 (3)	3 (1)
***Candida albicans***	2 (2)	1 (0.3)
***Cladosporium spp.^a^***	3 (3)	0
***A. albidus***	1 (1)	0
***Bactria^a^***	31 (31)	9 (3)
***A. terreus***	0	5 (1.7)
***Drechslera* spp.**	0	1 (0.3)
***Stachybotrys* spp.**	0	1 (0.3)
***Fusarium* spp.**	0	2 (0.7)
***Rhodotorula Rubra***	0	1 (0.3)

^a^ P Value ≤ 0.05

Table 2 shows the percentage of roasted and untreated nuts samples that were contaminated by fungi. In salty pistachios and peanuts, the most prevalent fungi were *A. fumigatus *(14%) and *Penicillium *spp. (14%) while in untreated peanuts, pistachios and walnuts, *A. niger *was predominant (14%, 62%, 41%, respectively). Percentages of *Aspergillus *genus were 54.3% and 35% for untreated samples and roasted with salt samples, respectively (P < 0.001). Bacterial contamination was 3% in untreated and 31% in roasted samples (P ≤ 0.001). There was a significant contamination in roasted with salt pistachio samples (50%). 

**Table 2.** Percentage of Prevalent Fungi Isolated From Untreated and Roasted With Salt Samples

Fungi	Untreated pistachios	Roasted pistachios	Untreated peanuts	Roasted peanuts	Untreated walnuts
***Aspergillus spp.***	79	24	19	46	71
***Mucor***	38	4	84	24	66
***Penicillium spp.***	22	2	13	14	41
***A. niger***	62	8	14	20	56
***A. flavus***	6	0	3	10	9
***A. fumigatus***	5	14	0	12	3
***A. ochraceus***	1	0	2	4	1
***Helminthosporium spp.***	39	0	6	6	15
***Acremonium spp.***	23	2	2	2	20
***Gliocladium spp.***	2	0	2	2	5
***Trichoderma spp.***	3	0	1	4	2
***Bactria***	5	50	0	12	4
***Other fungus***	5	8	2	0	3

According to Table 3, the most and the least mold contaminated nuts were walnuts (mean: 2.2 ± 0.98) and roasted with salt pistachios (mean: 0.38 ± 0.60), respectively. 

**Table 3.** Mean and Range of Fungi in Nuts

Nuts	Mean ± SD	Range
**Untreated walnuts**	2.2 ± 0.98	1 - 5
**Untreated pistachios**	2.1 ± 1.08	1 - 6
**Untreated peanuts**	1.29 ± 0.64	0 - 3
**Roasted pistachios**	0.38 ± 0.60	0 - 3
**Roasted peanuts**	0.98 ± 0.76	0 - 3

**Figure 1.**
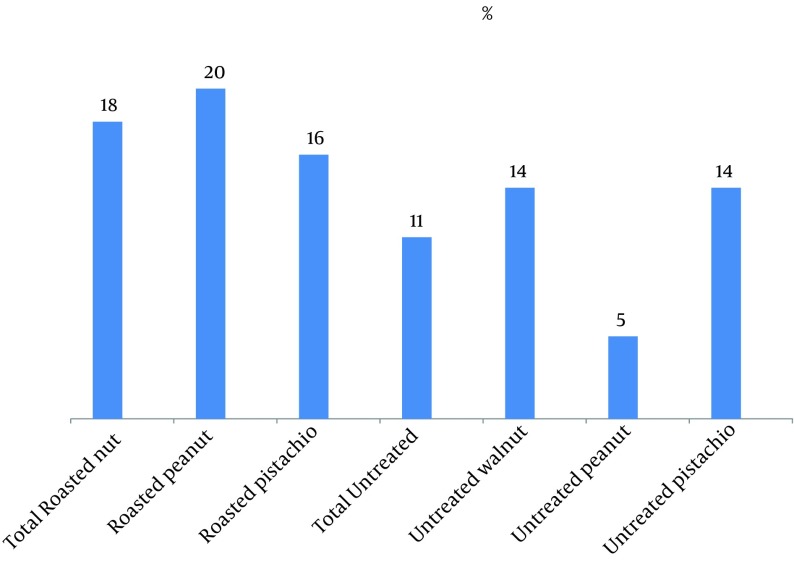
Percentage of Contamination With Mycotoxigenic Fungi The tested fungi include: *A. flavus*, *A. fumigatus*, *A. terreus*, *A. ochraceus*, *Penicillium* spp., *Gliocladium* spp., *Fusarium* spp., *Stachybotrys* spp.

According to Table 2, *Aspergillus *genus frequency was 79% in untreated pistachios and 71% in walnut samples, while contamination with this genus in untreated peanuts was 19%. *A. niger *was the predominant fungi in all untreated and roasted with salt peanut samples, while *A. fumigates *was prevalent in roasted with salt pistachios. Distinctive mycotoxigenic molds in the present study included *A. flavus *, *A. fumigatus *, *A. terreus *, *A. ochraceus *, *Penicillium *spp., *Gliocladium *spp., *Fusarium *spp., and *Stachybotrys *spp. and Figure 1 shows the contamination percentage of these fungi in samples. Percentage of mycotoxigenic fungal contamination in roasted samples (18%) was more than untreated samples (11%); this was considerable although not significant (P = 0.069). Fungal contamination in roasted samples was lower than untreated nuts. This reduction was significant for some types of fungi (P < 0.05) ( *Penicillium *spp., *A. niger *, *A. fumigatus *, *Acremonium *spp., *Helminthosporium *spp., *Cladosporium *spp., *A. ochraceus *). 

## 5. Discussion

Fungi accidently contaminate and decay food and food products (22). Fungal contamination of edible greasy seeds and nuts were reported from different countries (1). The obtained results show that the most dominant fungal genera in untreated and roasted with salt peanuts was *A. niger*, contaminating 14% of untreated and 20% of roasted with salt samples. In contrast to the findings of Hedayati, Gurses, Khomeiri, Vaamonde and Nakai, the present results showed that *A. niger* was prevalent in peanuts (both untreated and roasted with salt), whereas in their studies *A. flavus* was dominant (23-27). Furthermore, Hedayeti's study identified three strains of *Aspergillus* from peanut samples, namely *A. flavus*, *A. niger* and *A. fumigatus* (23). 

The results of another study on fungal contamination of peanuts in Zanjan Bazar (Iran) in comparison to Tabriz markets are shown in Table 4. Fungal contamination of peanuts in Tabriz is less than the Bazar of Zanjan (28). This can be the result of suitable conditions for the growth of fungi in the Bazar of Zanjan such as high temperature, high relative humidity, low light intensity, long-term storage and the natural fungal content of soil (29). Contamination of dried nuts such as peanuts, walnuts, and pistachios with mycotoxigenic species occurs generally during the harvest procedure and storage (24). 

**Table 4.** Percentage of Fungal Contamination of Zanjan and Tabriz Peanut Samples

Fungal Contamination	Zanjan		Tabriz	
	Untreated	Salted	Untreated	Salted
***A. niger***	62.5%	90%	14%	20%
***Penicillium*** ** spp.**	6.2%	40%	13%	14%
***A. flavus***	93.7%	60%	3%	10%

It seems that low fungal contamination in this study may be due to the nuts being fresh, because peanuts used in the present study were purchased during the harvesting season. Research on Egyptian peanuts showed that *A. niger* was the dominant mold in salted peanut samples (28.1%) which was similar to our results about roasted peanuts (20%); also in untreated samples *Penicillium* spp. (24%) was more than the present study (13%) (30). In the present study the predominant fungi in untreated pistachio samples were *Aspergillus* spp. (78%), *Helminthosporium* spp. (39%), *Acremonium* spp. (23%), *Penicillium* (22%) and *Mucor* (38%), while in roasted with salt pistachios, *Aspergillus* spp. (24%) and *Cladosporium* spp. (16%) were prevalent. Among the *Aspergillus* family, *A. niger* with 62% contamination was prevalent in untreated pistachios, but in roasted with salt pistachios, *A. fumigatus* with 14% contamination was dominant. 

The isolation of *Aspergillus* spp. agrees with the findings of other researchers evaluating peanuts in tropical areas (31, 32). The prevalence of *Aspergillus* may be because of the climatic conditions of these countries, which can be appropriate for the development of fungi like *Aspergillus* (33). A study by Fernane reported that contamination by *A. niger* (34) was 30% which is less than Tabriz untreated and roasted with salt pistachios with 62% and 8% contamination rate, respectively. Furthermore, as reported by Fernane, pistachios contamination by *Penicillium* spp. was 38% and more than untreated and roasted with salt pistachios contamination in samples of Tabriz markets. Also Shahidi reported that 12.5% of pistachio samples were contaminated by *Aspergillus* species (35). The results of another study on pistachios fungal contamination in America showed that *Aspergillus* spp. with 94.5% contamination rate was the dominant fungi (36). 

*A. niger* was the most frequently isolated mold in walnuts, contaminating 56% of walnut samples. Walnut contamination by this fungus was less than that of Saudi Arabia (78.5%) (13). The excessive growth of *Aspergillus* spp. may be due to the fact that this genus is regarded as a storage mold while *Fusarium* spp. is considered as a field fungus (33). High fungal contamination in walnuts may be due to the fact that, all walnut samples were without shells (12). Also some samples had been damaged, thus this factor beside others mentioned above may facilitate the fungal growth on nuts (7, 27, 28).

 Some explanations for the low contamination of roasted samples can be suggested. Firstly, salt on nuts is an important factor which prevents the growth of different fungi (37, 38). Secondly, low moisture content of salted and roasted nuts is a preventive factor of mold growth. Thirdly, it seems that high temperature during roasting of nuts can reduce fungal contamination of nuts (39, 40). Results of this study showed that contamination with *Mucor* was prevalent especially in untreated nuts. Even though this fungus is not a mycotoxigenic fungi, contamination with this microorganism should be monitored for maintenance of foodstuff hygiene and safety. Furthermore, it is suggested that high microbial contamination of roasted with salt samples should be further studied.

According to the results of the present study, incidence of fungal contamination in nuts with *Aspergillus* family and mycotoxigenic molds is high; on the other hand, nuts are considered as an Iranian favorite snack due to their notable health effects. Since consumption of contaminated nuts for long periods has carcinogenic and toxigenic effects on human health, hence government authorities should monitor and set suitable guidelines for food safety, furthermore take steps to educate people about the dangers of toxigenic molds and promote the health knowledge for people to select safe foods.

## References

[1] Reza Khosravi A, Shokri H, Ziglari T (2007). Evaluation of Fungal Flora in Some Important Nut Products (Pistachio, Peanut, Hazelnut and Almond) in Tehran, Iran.. Pak J Nutr..

[2] Horn BW (2005). Colonization of wounded peanut seeds by soil fungi: selectivity for species from Aspergillus section Flavi.. Mycologia..

[3] Kacaniova M (2003). Feeding soybean colonization by microscopic fungi.. Trakya Univ J Sci..

[4] Kazemi A (April 24-25, 2003). Carcinogenic mycotoxins. The 1st Student's Congress of Cancer.

[5] Soubra L, Sarkis D, Hilan C, Verger P (2009). Occurrence of total aflatoxins, ochratoxin A and deoxynivalenol in foodstuffs available on the Lebanese market and their impact on dietary exposure of children and teenagers in Beirut.. Food Addit Contam Part A Chem Anal Control Expo Risk Assess..

[6] Wang J, Liu XM (2007). Contamination of aflatoxins in different kinds of foods in China.. Biomed Environ Sci..

[7] Bayman P, Baker JL, Mahoney NE (2002). Aspergillus on tree nuts: incidence and associations.. Mycopathologia..

[8] Rezaeian F, Zamene Milani F, Kazemi A, Mohtadi Nia J, Ghaem Maghami SJ, Jabbari M (4-7 September 2006). Contamination of tea and traditional vegetable distilled to mycotoxin producer fungi. 9th Iranian Nutrition Congress.

[9] (2000.). Safety evaluation of certain mycotoxins in food.

[10] Weidenborner M (2001). Pine nuts: the mycobiota and potential mycotoxins.. Can J Microbiol..

[11] Gangneux JP, Noussair L, Bouakline A, Roux N, Lacroix C, Derouin F (2004). Experimental assessment of disinfection procedures for eradication of Aspergillus fumigatus in food.. Blood..

[12] Luttfullah G, Hussain A (2011). Studies on contamination level of aflatoxins in some dried fruits and nuts of Pakistan.. Food Cont..

[13] Alwakeel SS, Nasser LA (2011). Microbial Contamination and Mycotoxins from Nuts in Riyadh, Saudi Arabia.. Am J Food Technol..

[14] Campbell BC, Molyneux RJ, Schatzki TF (2003). Current research on reducing pre-and post-harvest aflatoxin contamination of US almond, pistachio, and walnut.. Toxin Rev..

[15] Kubatova A [New species of toxinogenicPenicillium found in the foods and their identification]. [Current Problems of Food Microbiology II]..

[16] Dorner JW (2008). Management and prevention of mycotoxins in peanuts.. Food Addit Contam Part A Chem Anal Control Expo Risk Assess..

[17] Kashaninejad M, Mortazavi A, Safekordi A, Tabil LG (2006). Some physical properties of Pistachio (Pistacia vera L.) nut and its kernel.. J Food Eng..

[18] Bankole SA, Ogunsanwo BM, Eseigbe DA (2005). Aflatoxins in Nigerian dry-roasted groundnuts.. Food Chem..

[19] Sabate J, Ang Y (2009). Nuts and health outcomes: new epidemiologic evidence.. Am J Clin Nutr..

[20] Booth C (1971). The Genus Fusarium..

[21] Samson RA, Hoeckstra VR, Frisvad JC, Filtenborg O (2002). Introduction to Food and Airborne Fungi..

[22] Kazemi A, Mohtadi Nia J, Mahdavi R, GhaemMaghami SJ, Akbari N, Salehpour A (2008). Mycotoxicogenic fungal contamination of consumed rice in east Azarbaidjan.. J Tabriz Univ Med Sci..

[23] Hedayati MT, Pasqualotto AC, Warn PA, Bowyer P, Denning DW (2007). Aspergillus flavus: human pathogen, allergen and mycotoxin producer.. Microbiology..

[24] Gürses M (2006). Mycoflora and aflatoxin content of hazelnuts, walnuts, peanuts, almonds and roasted chickpeas (LEBLEBI) sold in Turkey.. Int J Food Prop..

[25] Khomeyri M, Maghsoudlou Y, Kumar L, Vali Yar F, Hasani S (2008). Determination of AF contamination and Aspergillusflavus infection of ground nuts from northern provinces of Iran.. J Agr Sci Nat Res..

[26] Vaamonde G, Patriarca A, Fernandez Pinto V, Comerio R, Degrossi C (2003). Variability of aflatoxin and cyclopiazonic acid production by Aspergillus section flavi from different substrates in Argentina.. Int J Food Microbiol..

[27] Nakai VK, de Oliveira Rocha L, Gonçalez E, Fonseca H, Ortega EMM, Corrêa B (2008). Distribution of fungi and aflatoxins in a stored peanut variety.. Food Chem..

[28] Rostami R, Naddafi K, Aghamohamadi A (2009). Survey Of Peanut Fungal Contamination And Its Relationship With Ambient Conditions In The Bazar Of Zanjan.. Iran J Env Health Sci Eng..

[29] Pitt JI, Dyer SK, McCammon S (1991). Systemic invasion of developing peanut plants by Aspergillus flavus.. Lett Appl Microbiol..

[30] Youssef MS, El-Maghraby OMO, Ibrahim YM (2008). Mycobiota and Mycotoxins of Egyptian Peanut (Arachis hypogeae L.) Seeds.. Int J Botan..

[31] Bhattacharya K, Raha S (2002). Deteriorative changes of maize, groundnut and soybean seeds by fungi in storage.. Mycopathologia..

[32] Njobeh PB, Dutton MF, Koch SH, Chuturgoon A, Stoev S, Seifert K (2009). Contamination with storage fungi of human food from Cameroon.. Int J Food Microbiol..

[33] Hedayati MT, Kaboli S, Mayahi S (2010). Mycoflora of pistachio and peanut kernels from Sari, Iran.. Jundishapur J Microbiol..

[34] Fernane F, Sanchis V, Marin S, Ramos AJ (2010). First report on mould and mycotoxin contamination of pistachios sampled in Algeria.. Mycopathologia..

[35] Shahidi Bonjar GH (2004). Incidence of Aflatoxin Producing Fungi in Early Split Pistachio Nuts of Kerman, Iran.. J Biol Sci..

[36] Doster MA, Michailides TJ, Goldhamer DA, Morgan DP (2001). Insufficient spring irrigation increases abnormal splitting of pistachio nuts.. California Agr..

[37] Kilcast D, Subramaniam P (2000). Introduction in the stability and shelf-life of food..

[38] Raei M, Mortazavi A, Pourazarang H (2009). Effects of Packaging Materials, Modified Atmospheric Conditions, and Storage Temperature on Physicochemical Properties of Roasted Pistachio Nut.. Food Anal Methods..

[39] Nawar LS (2008). Prevention and control of fungi contaminated stored pistachio nuts imported to Saudi Arabia.. Saudi J Biol Sci..

[40] Cheraghali AM, Yazdanpanah H, Doraki N, Abouhossain G, Hassibi M, Ali-abadi S (2007). Incidence of aflatoxins in Iran pistachio nuts.. Food Chem Toxicol..

